# Supplementary material for: Structural basis of excitatory amino acid transporter 3 substrate recognition
Source: Proc Natl Acad Sci U S A. 2025 Apr 18;122(16):e2501627122. doi: 10.1073/pnas.2501627122 (PMC12036983; doi:10.1073/pnas.2501627122)
Supplement: Supplementary file 1 — Appendix 01 (PDF) [file pnas.2501627122.sapp.pdf]

Supporting information for

Structural basis of excitatory amino acid transporter 3 substrate recognition.

Biao Qiu[1,2], Olga Boudker[1,2, *]

[1] Department of Physiology & Biophysics, Weill Cornell Medicine, 1300 York Ave, New York, NY 10021, USA

[2] Howard Hughes Medical Institute, Weill Cornell Medicine, 1300 York Ave, New York, NY 10021, USA

[*] Correspondence: olb2003@med.cornell.edu

This PDF file **includes**

Figures S1 to S12

Tables S1

Legend for Movie S1

Other supporting materials for this manuscript include the following:

Movie S1

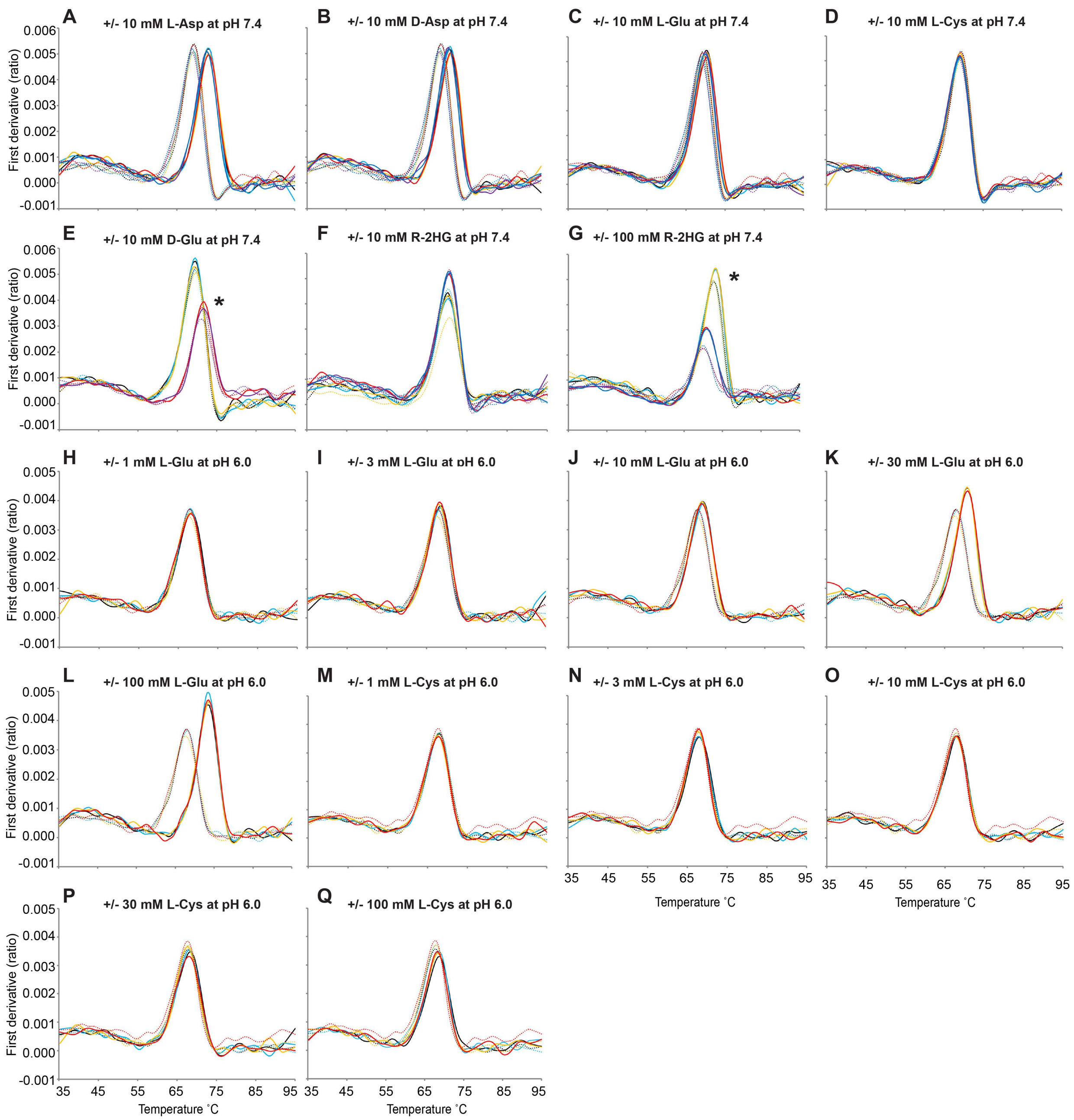

Fig. S1. Raw thermal melt data for hEAAT3g in the absence and presence of substrates. The fluorescence emission ratio at 350 and 330 nm was measured as a function of increasing temperature, and the first derivatives of these data are shown in the panels. Dotted lines represent data obtained in the presence of 200 mM Na^+ without any substrate; solid lines correspond to data in the presence of the specified concentration of each substrate. Melting temperature (Tm) is calculated as the temperature at the peak of the first derivative trace, which corresponds to the inflection points of the melting curve. The Tm increases (ΔTm-s) are differences in Tm-s observed in the presence and absence of substrates. All measurements were conducted on at least two independently prepared protein samples. (**A-G**), Traces for ΔTm calculation for 10 mM L-Asp, D-

Asp, L-Glu, L-Cys, D-Glu, R-2HG, and 100 mM R2-HG at pH 7.4. (**H-Q**), Traces for ΔTm calculation (**Fig. 1E**, left) for 1, 3, 10, 30, and 100 mM L-Glu (**H-L**) and L-Cys (**M-Q**) at pH 6.0. An asterisk (*) indicates instances where two independent protein preparations exhibited slightly different Tm values, possibly due to minor buffer differences; however, the measured ΔTm values were consistent across preparations. **Fig. 1B** shows the fifth measurements from (**A-D**) as examples of the raw data. **Fig. 1C** was assembled using data from panels (**A-G**) and (**Q**).

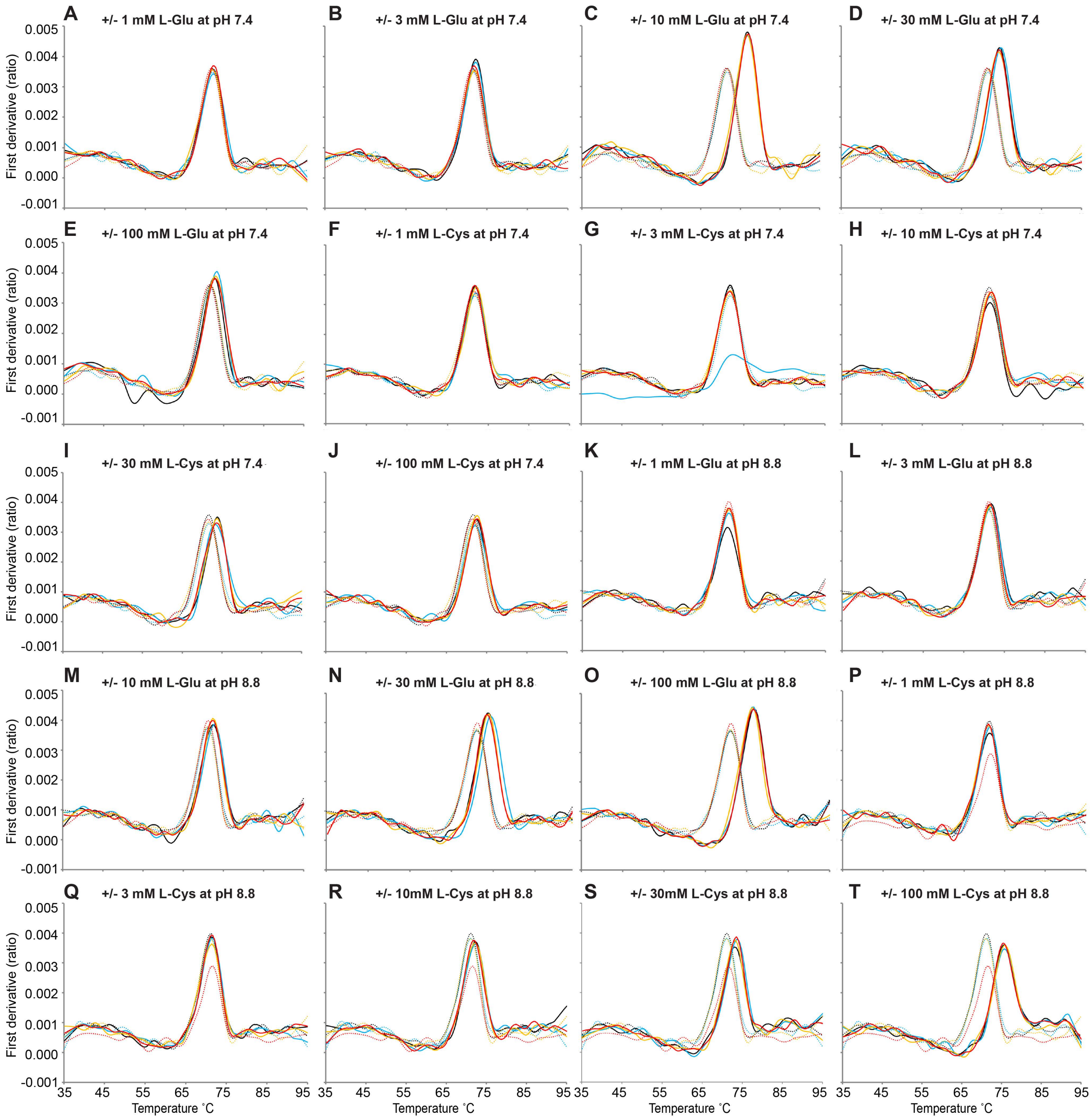

Fig. S2. Same as Fig. S1. Traces for ΔTm calculation (**Fig. 1E**, middle and right) for 1-100 mM L-Glu at pH 7.4 (**A-E**) and 8.8 (**K-O**) and 1-100 mM L-Cys at pH 7.4 (**F-J**) and 8.8 (**P-T**). The ΔTm values from panels (**J**) and (**T**) were included in **Fig. 1C**.

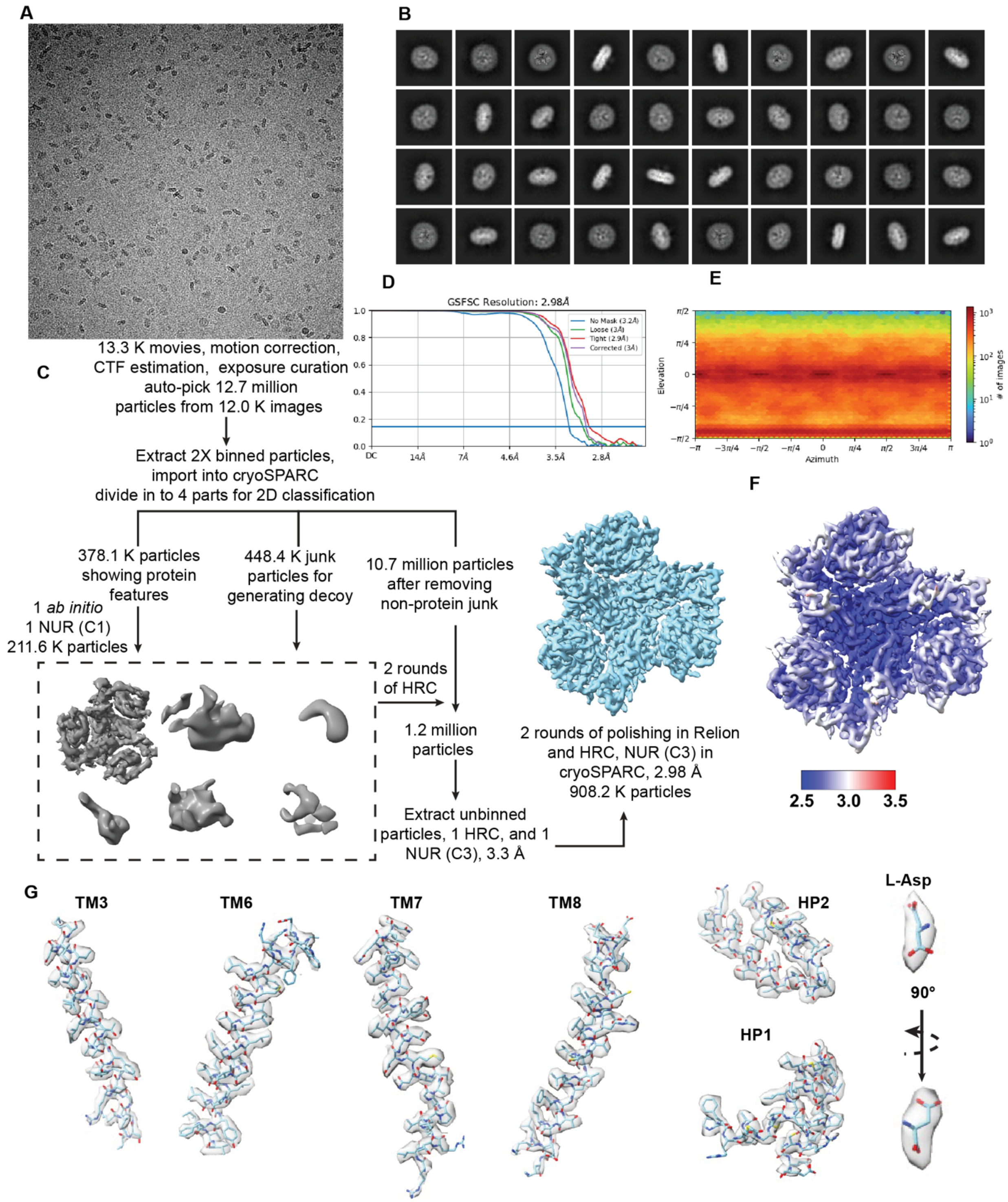

Fig. S3. Cryo-EM analysis of hEAAT3-X in complex with L-Asp. A representative image (**A**) and selected 2D class averages (**B**) of the L-Asp dataset. (**C**), The cryo-EM data processing flow. (**D**), The golden standard Fourier shell correlation (FSC) curves of the final refinement. (**E**), The angular distribution of particles used for the final 3D reconstitutions. (**F**), The local resolution distribution of the final map. (**G**), The EM density of L-Asp, transport domain transmembrane

helices (TMs), and helical hairpins (HPs); the map contour level is 0.813 in ChimeraX, corresponding to 5σ.

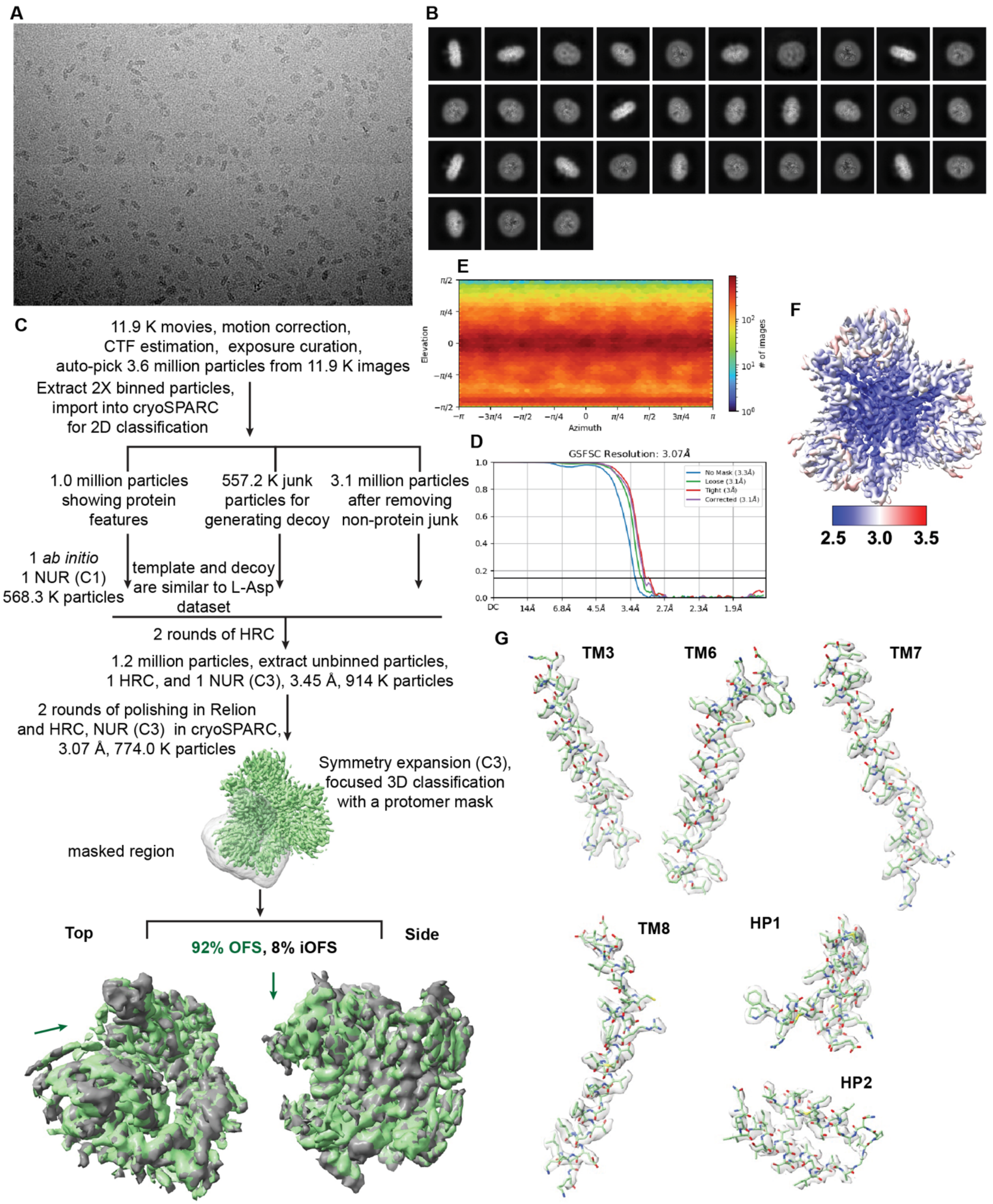

Fig. S4. Cryo-EM analysis of hEAAT3-X with R-2HG. A representative image (**A**) and selected 2D class averages (**B**) of the R-2HG dataset. (**C**), The cryo-EM data processing flow. The template and decoy volumes, similar to those in **Fig. S3**, are not shown for clarity; the green arrows at the bottom show the transport domain movement from OFS to iOFS. (**D**), The FSC curves of the final refinement. (**E**), The angular distribution of the particles used for the final 3D reconstitutions. (**F**),

The local resolution distribution of the final map. (**G**), The EM density of the transport domain TMs and HPs; the map contour level is 0.34 in ChimeraX, corresponding to 5σ. The molecular model of Na^+-only bound EAAT3-X (PDB: 8CV2) was fitted into the final map without refinement for reference.

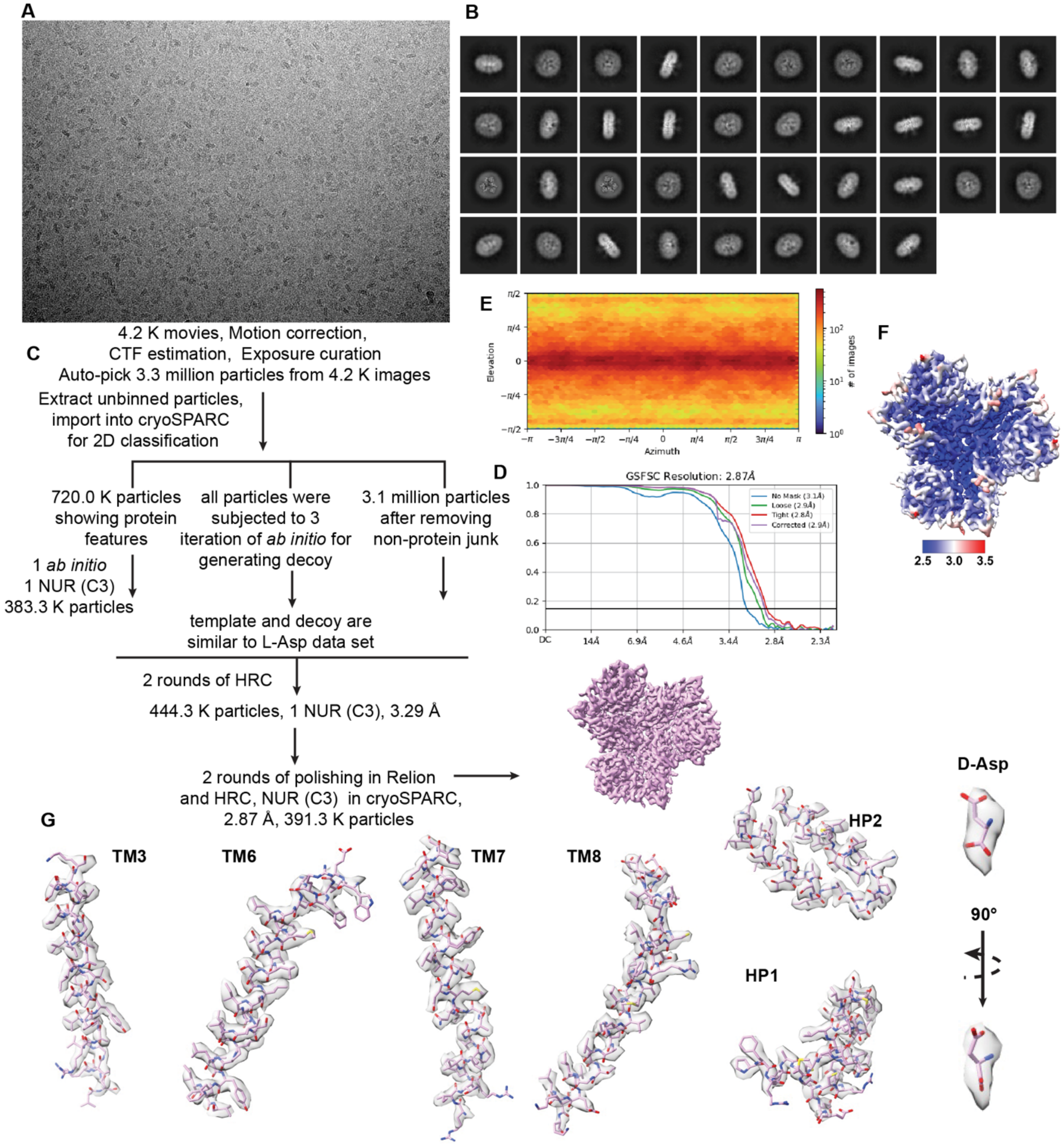

Fig. S5. Cryo-EM analysis of hEAAT3-X in complex with D-Asp. A representative image (**A**) and selected 2D class averages (**B**) of the D-Asp dataset. (**C**), The cryo-EM data processing flow. (**D**), The FSC curves of the final refinement. (**E**), The angular distribution of particles used for the final 3D reconstitutions. (**F**), The local resolution distribution of the final map. (**G**), The EM density of D-Asp, the TMs, and HPs; the contour level is 0.907 in ChimeraX, corresponding to 5σ.

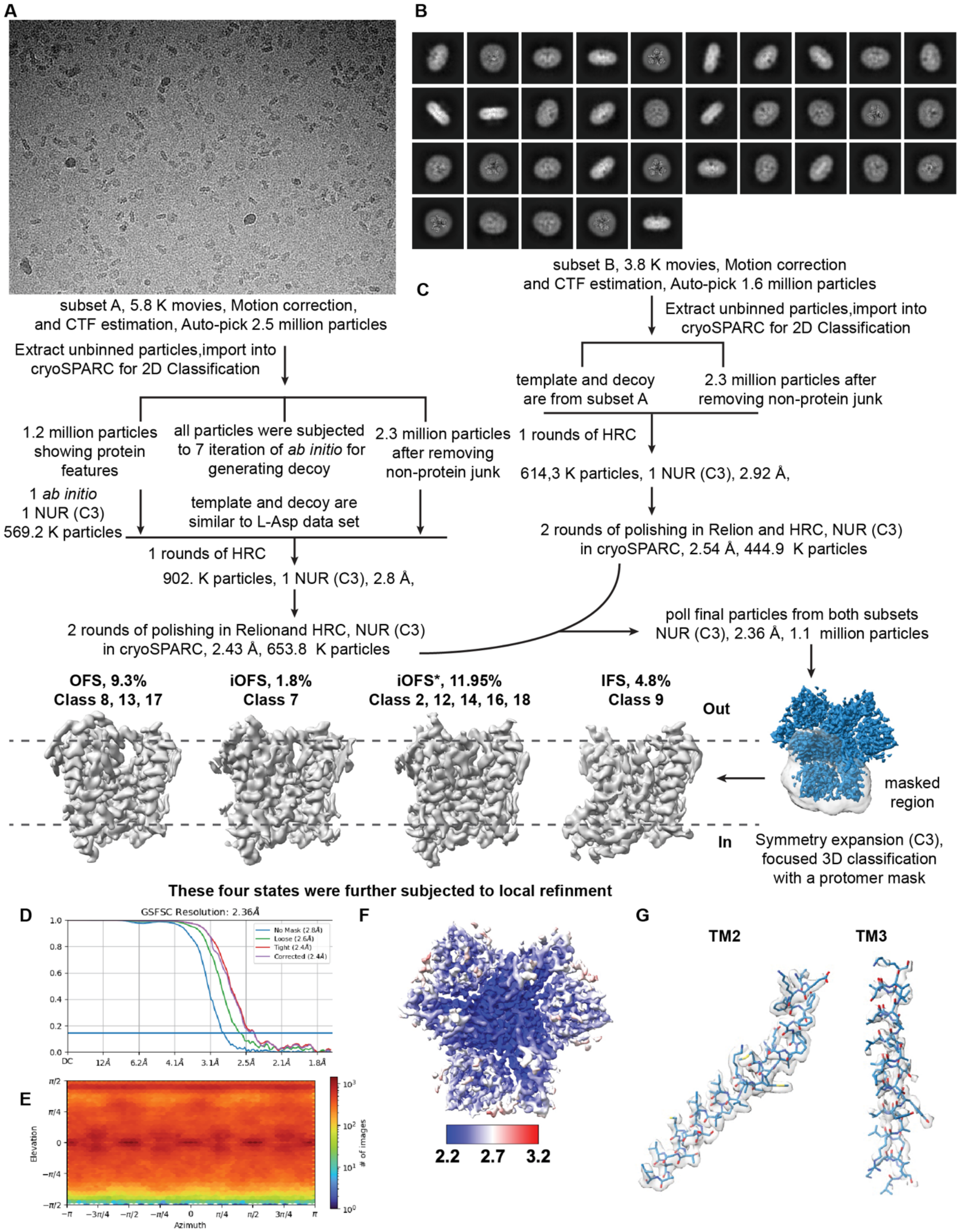

Fig. S6. Cryo-EM analysis of hEAAT3-X in complex with L-Cys. A representative image (**A**) and selected 2D class averages (**B**) of the L-Cys dataset. (**C**), The cryo-EM data processing flow. The protomer's reconstitution information is shown in **Fig. S7, 8**. (**D**), The FSC curves of the trimer

map. (**E**), The angular distributions of particles used for the 3D reconstitutions of the trimer. (**F**), Local resolution distribution of the trimer map. (**G**), The EM density of TM2 in the scaffold domain and TM3 in the transport domain with the contour level of 0.62 in ChimeraX, corresponding to 5σ; the blurred density of TM3 reflects the dynamics of the transport domain.

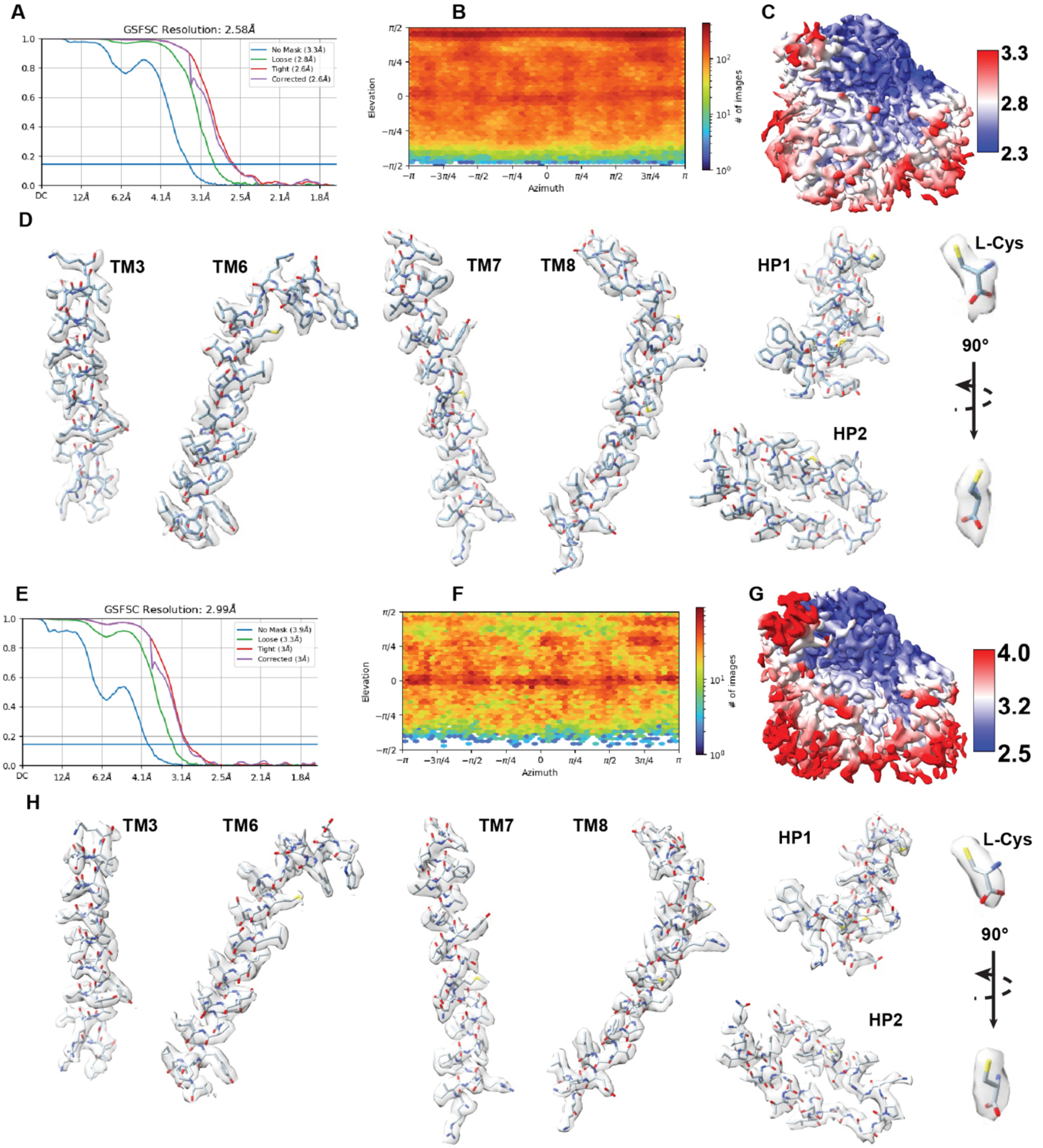

Fig. S7. The local refinement of hEAAT3-X in complex with L-Cys in OFS and iOFS. The FSC curves of the OFS-L-Cys (**A**) and iOFS-L-Cys (**E**). The angular distribution of particles used for the 3D reconstitutions of OFS-L-Cys (**B**) and iOFS-L-Cys (**F**). The local resolution distribution of OFS-L-Cys map (**C**) and iOFS-L-Cys map (**G**). The EM density of L-Cys, transport domain TMs, and HPs of OFS-L-Cys (**D**) and iOFS-L-Cys (**H**). The map contour levels in (**D**) and (**H**) are 0.65 and 0.54, respectively, in ChimeraX, corresponding to 3.3σ.

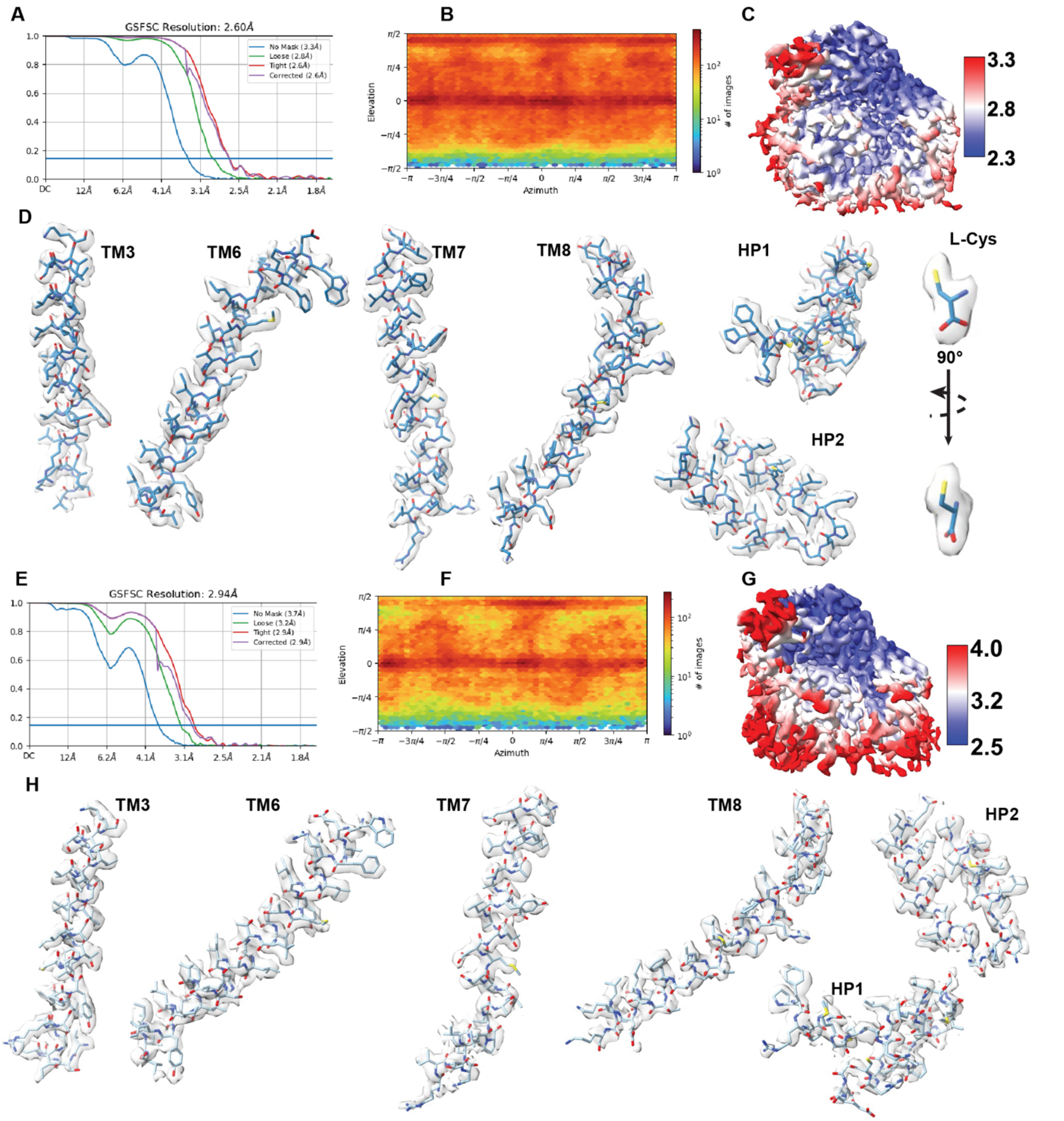

Fig. S8. The local refinement of hEAAT3-X in complex with L-Cys in iOFS* and sodium-only bound IFS (IFS-Na$^+$). The FSC curves of the iOFS*-L-Cys (**A**) and IFS-Na$^+$ (**E**). The angular distribution of particles used for the 3D reconstitutions of iOFS*-L-Cys (**B**) and IFS-Na$^+$ (**F**). The local resolution distribution of iOFS*-L-Cys (**C**) and IFS-Na$^+$ (**G**). The EM density of L-Cys and transport domain TMs and HPs of iOFS*-L-Cys (**D**) and IFS-Na$^+$ (**H**). The map contour levels in (**D**) and (**H**) are 0.61 and 0.43, respectively, in ChimeraX, corresponding to 3.3σ. The IFS-Na$^+$ molecular model (PDB: 6X2L) was fitted in the IFS-Na$^+$ map without refinement for reference.

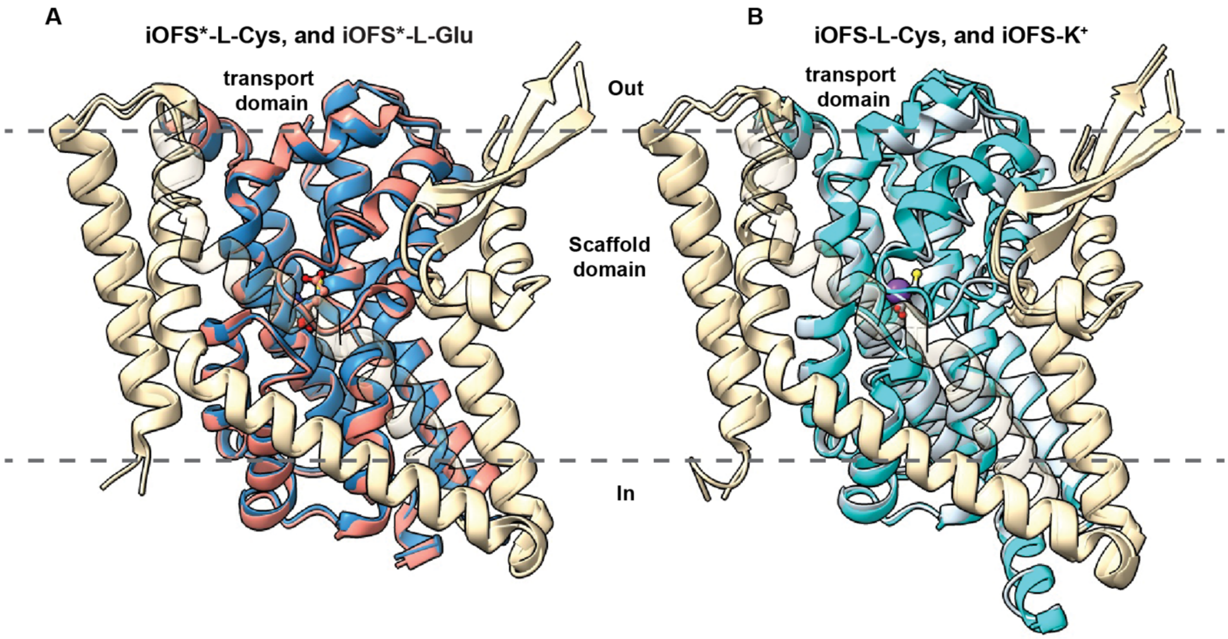

Fig. S9. The overall structures of iOFS*-L-Cys and iOFS-L-Cys. (**A, B**), Superpositions over the entire protomers of (**A**) iOFS*-L-Cys (blue) and iOFS*-Glu (salmon, PDB: 8CTC) and (**B**) iOFS-L-Cys (light pastel blue) and iOFS-K$^+$ (cyan, PDB: 8CUA). The scaffold domains are colored in wheat, and TM2 is rendered transparent for clarity. The bound L-Glu, L-Cys, and potassium ion are shown as ball-and-stick models and a sphere.

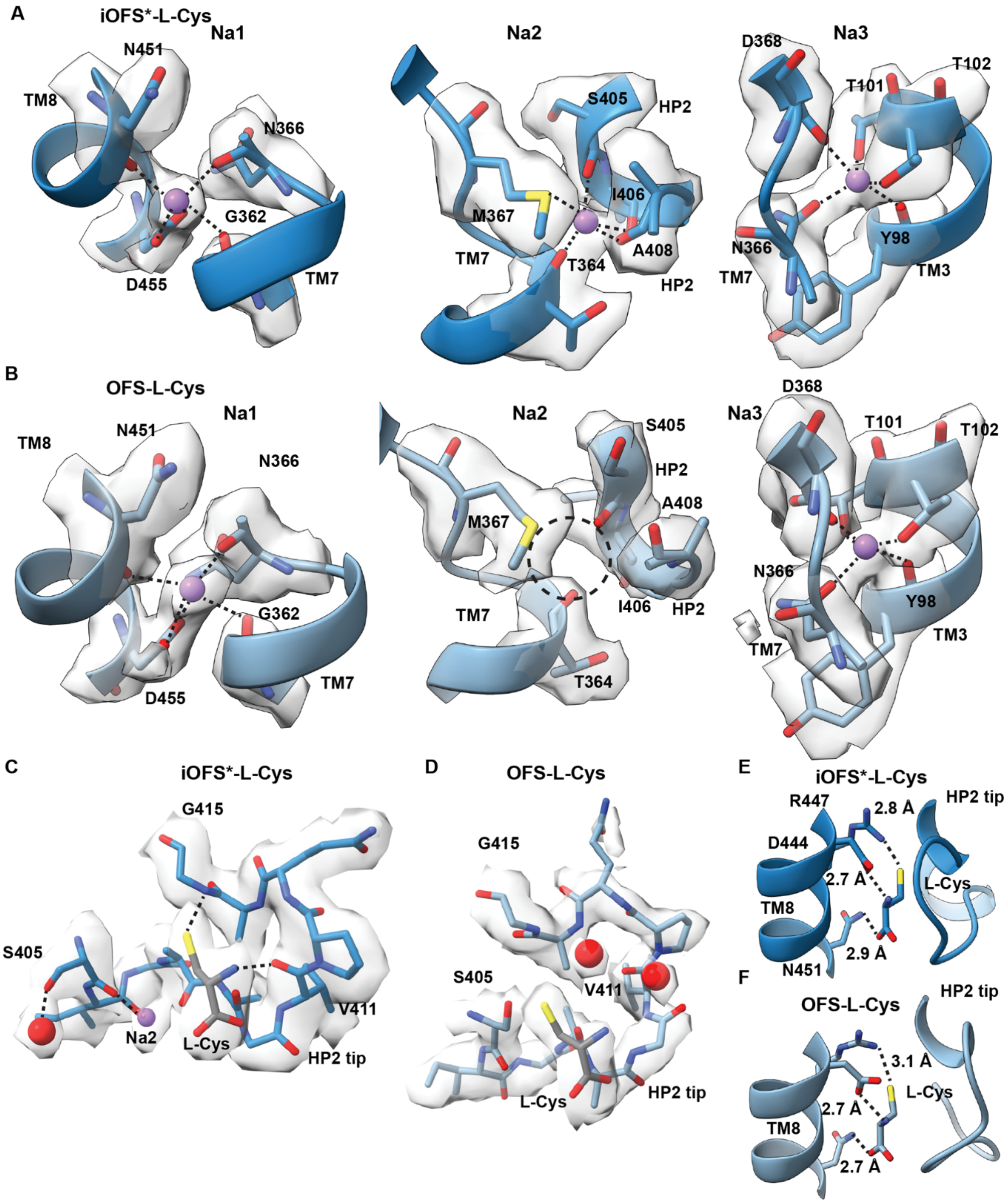

Fig. S10. The EM density at sodium-binding sites and the structure of HP2 tip in iOFS*-L-Cys (dark blue) and OFS-L-Cys (pastel blue). The EM density and geometry of sodium sites in iOFS*-L-Cys (**A**) and OFS-L-Cys (**B**). From left to right: Na1, Na2, and Na3 sites. The dashed circle highlights the distorted Na2 site in OFS-L-Cys. (**C**), The interactions between L-Cys thiolate and the HP2 tip in iOFS*-L-Cys are highlighted as dashed lines. (**D**), They are disrupted in OFS-L-Cys, with water molecules (red spheres) entering the enlarged space. Interactions between L-Cys and coordinating amino acids in TM8 are similar in iOFS*-L-Cys (**E**) and OFS-L-Cys (**F**).

Na^+ ions and water molecules are shown as spheres scaled to 0.5-fold of their van der Waals radii. The contour levels of density maps in iOFS*-L-Cys and OFS-L-Cys are 0.6 and 0.5, corresponding to 3.3σ and 2.6σ, respectively. The dashed black lines show the interactions between protein residues and ligands.

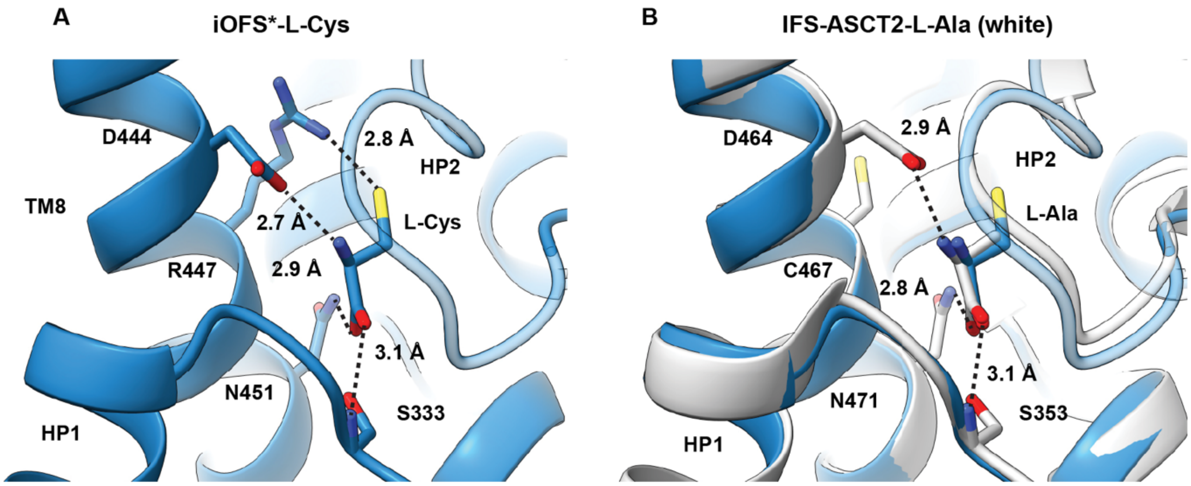

Fig. S11. L-Cys recognition in EAAT3 and ASCT2. (**A, B**), The dashed black lines show the interaction of the residues in TM8 with substrates in EAAT3 (**a,** dark blue), and ASCT2 (**B,** PDB: 8OUD, white). The superposition (**B**) shows nearly identical protein structures and similar poses of bound L-Cys in EAAT3 and L-Ala in ASCT2. C467 in ASCT2 is at the equivalent position to R447 in EAAT3; it is likely too far to interact with bound L-Cys. The structures are superposed on the cytoplasmic halves of their transport domains (residues 314-372 and 442-465 in EAAT3 and residues 334-392, 462-485 in ASCT2).

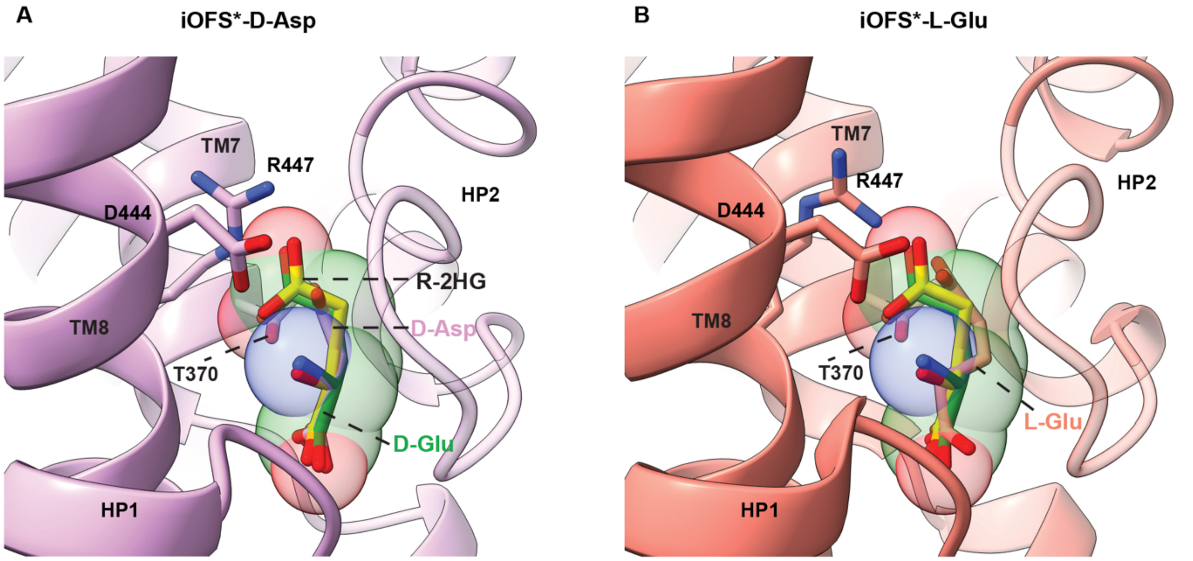

Fig. S12. Models of D-Glu and R-2HG in EAAT3 substrate-binding site. The Cα, α carboxyl, and amino groups of the D-Glu (green stick and semitransparent spheres) and the Cα, α carboxyl, and hydroxyl groups of R-2HG (yellow sticks) were modeled based on the Cα, α carboxyl, and amino groups of D-Asp (pink sticks, **A**). The models show that the γ carboxyl of D-Glu and R-2HG may cause steric clashes with R447 and T370 in the ligand binding pocket, unlike D-Asp (pink, **A**) and L-Glu (salmon, **B**, PDB: 8CTC). The structures are superposed on the cytoplasmic halves of their transport domains (residues 314-372 and 442-465).

Supplementary Movie 1: The partial and complete HP2 gate closure upon substrate and Na2 binding. The protein model colors, and coordinate files are the same as in **Fig. 5**. Na1 and Na3 are not shown for clarity. The cytoplasmic halves of the transport domains (residues 314-372 and 442-465) were used for superposition and morphing.

Table S1. Cryo-EM data collection, processing, and model refinement Statistics

	iOFS*-L-Asp	OFS-R-2HG (Na^+ only)	iOFS*-D-Asp	OFS-L-Cys	iOFS-L-Cys	iOFS*-L-Cys	IFS-Cys (Na^+ only)
**Data collection and processing**							
Magnification	100,100 X	100,500 X	64,000 X	100,500 X			
Voltage (kV)	200	300	300	300			
Electron exposure (e–/Å²)	40	50.54	52.19	58.25			
Defocus range (μm)	-1.0 – -2.5	-0.8 – -2.2	-0.5 – -2.0	-0.8 – -2.4			
Pixel size (Å)	1.16	0.844	1.076	0.825			
Symmetry imposed	C3	C3	C3	C3 for initial trimer, C1 for expanded protomer			
Initial particle images (no.)	12,668,720	3,6262,598	3,346,010	4,180,263 initial; 1,112,764 final; 3,338,292 C3 expanded			
Final particle images (no.)	908,281	773,970	391,308	307,042	60,670	365,660	159,538
Map resolution (Å)	2.98	3.07	2.87	2.58	2.99	2.60	2.94
FSC threshold	0.143	0.143	0.143	0.143	0.143	0.143	0.143
Map resolution range (Å)	7.42 – 2.56	7.19 – 2.58	36.71 – 2.43	41.24 – 2.31	38.18 – 2.65	30.80 – 2.35	42.46 – 2.58
**Refinement**		This map is					This map is
Initial model used (PDB code)	8CTC	similar to	8CTC	6X2Z	8CV3	8CTC	similar to
Model resolution (Å)	3.0	EMD-27006	2.9	2.8	3.3	2.7	EMD-22011
FSC threshold	0.5	(EAAT3-X,	0.5	0.5	0.5	0.5	(EAAT3, IFS-
Map sharpening *B* factor (Å²)	-164	OFS-Na^+).	-116				Na^+). The
Model composition		The model					model 6X2L
Non-hydrogen atoms	9,306	8CV2 can be	9,297	3,242	3,143	3,105	can be fitted
Protein residues	1,221	fitted in this	1,218	426	413	407	in this map.
Ligands	15	map.	15	3	5	5	
B factors (Å²)							
Protein	51.23		65.75	40.46	59.17	38.93	
Ligand	88.27		62.08	41.15	104.00	70.93	
R.m.s. deviations							
Bond lengths (Å)	0.005		0.004	0.004	0.004	0.004	
Bond angles (°)	1.078		0.979	0.934	0.953	0.904	
Validation							
MolProbity score	1.13		1.27	1.12	1.17	1.10	
Clashscore	3.35		4.81	3.30	3.87	3.14	
Poor rotamers (%)	0.00		0.00	0.00	0.00	0.00	
Ramachandran plot							
Favored (%)	98.25		97.92	99.05	98.28	98.25	
Allowed (%)	1.75		2.08	0.95	1.72	1.75	
Disallowed (%)	0.00		0.00	0.00	0.00	0.00	
**PDB code**	9D66		9D67	9D68	9D69	9D6A	
**EMDB code**	46586	46587	46588	46589	46590	46591	46592
